# Efficacy of ursolic acid against *Echinococcus granulosus* in vitro and in a murine infection model

**DOI:** 10.1186/s13071-018-2628-8

**Published:** 2018-01-24

**Authors:** Jianhai Yin, Congshan Liu, Yujuan Shen, Haobing Zhang, Jianping Cao

**Affiliations:** 1 National Institute of Parasitic Diseases, Chinese Center for Disease Control and Prevention, Shanghai, 200025 China; 2 Key Laboratory of Parasite and Vector Biology, MOH, Shanghai, 200025 China; 3 National Center for International Research on Tropical Diseases, Shanghai, 200025 China; 4 WHO Collaborating Centre for Tropical Diseases, Shanghai, 200025 China

**Keywords:** *Echinococcus granulosus*, Ursolic acid, Protoscoleces, Germinal cells, Metacestodes, Ultrastructural damage

## Abstract

**Background:**

Cystic echinococcosis is a global public health problem; however, the drugs (albendazole and mebendazole) currently recommended by WHO for its treatment, have limited efficacy. Therefore, novel drugs are required to provide more choices for the treatment of this disease.

**Methods:**

The anthelmintic effects of ursolic acid (UA) were tested on *Echinococcus granulosus* protoscoleces, germinal cells and metacestodes in vitro. The in vivo efficacy of UA was investigated in mice following secondary infection with *E. granulosus*. Furthermore, the corresponding ultrastructural damage induced by UA was evaluated by electron microscopy.

**Results:**

In vitro, 45.95 ± 5.30% of protoscoleces were killed by UA at 40 μg/ml, while the growth of more than 90% of germinal cells was inhibited by UA at 10 to 40 μg/ml. The same effect was observed in metacestodes 7 days after treatment with UA at 10, 20 and 40 μg/ml, and more than 50% of metacestodes showed loss of integrity at the end of the experiment. In vivo, metacestode weight was significantly reduced following oral administration of UA at 200 and 100 mg/kg (39.5 and 38.3%, respectively). Additionally, ultrastructural damage, such as alternations in germinal cell morphology and formation of vacuoles and lipid granules were observed in parasites treated with UA in vitro, while detachment of the germinal layer from the laminated layer was also seen in metacestodes in vivo.

**Conclusions:**

UA was demonstrated to exert parasiticidal activity against *E. granulosus* in vitro and in vivo, thus implicating UA as a potential anti-echinococcosis agent.

## Background

Cystic echinococcosis (CE), also known as hydatid disease, which is caused by taeniid cestodes *Echinococcus granulosus* (*sensu lato*) in the larval stage, represents an important public health problem due to its wide geographical distribution as well as its medical and economic impact [1]. China is considered to be one of the most important endemic regions of this disease [2]. The life-cycle of *E. granulosus* involves dogs and other canids as definitive hosts, and numerous mammalian species, including domestic livestock and humans as intermediate hosts. Once the eggs are ingested by humans, hydatid cysts may develop, mostly in the liver and lungs. Despite its importance with regard to health, there are only two drugs (albendazole and mebendazole) available for the treatment of echinococcosis, both exhibiting some adverse effects and limited efficacy in the chronic stage of the disease [3]. Therefore, the development of novel drugs to provide more choices for the treatment of CE is necessary.

To date, several drugs that inhibit cancer cell proliferation have been evaluated for their effects on *Echinococcus* spp. based on the similarities between cancer cells and parasites. Tamoxifen, which was widely used for treating primary breast cancer, inhibited the survival of *E. granulosus* protoscoleces and metacestodes at 10 to 50 μM, and a reduction in cyst weight was observed following the administration of 20 mg/kg tamoxifen [4]. The proteasome inhibitor, bortezomib, which was developed for myeloma chemotherapy, displays high activity against *E. multilocularis* metacestodes in vitro although the activity of this drug was lower than that of albendazole and induced adverse effects in vivo [5]. Furthermore, 5-fluorouracil and paclitaxel [6], as well as imatinib [7], exhibit profound anti-parasitic activity against *E. multilocularis*. These results suggest that novel candidate drugs for the treatment of CE might be found among the chemicals with antitumor effects.

In the present study, we investigated the efficacy of ursolic acid (UA) against *E. granulosus*. As an ursane-type pentacyclic triterpenic acid, UA is ubiquitous in the leaves and berries of natural medicinal plants [8, 9]. UA induces apoptosis and inhibits proliferation or growth in many types of malignancies both in vivo and in vitro [10–12]. UA has been developed as a liposome formulation for the treatment of cancer and the phase I trials demonstrated tolerable toxicity and adverse effects [13]. UA was also reported to have biological potential as an antibacterial [14] and antiviral [15] agent. Furthermore, the antiparasitic activity of UA has been demonstrated in protozoa [16] and helminths [17–19].

In this study, we evaluated the in vitro anthelmintic effects of UA on protoscoleces, germinal cells and metacestodes of *Echinococcus granulosus*. Moreover, the in vivo efficacy and cytotoxicity of UA were also investigated in experimentally infected mice.

## Methods

### Chemicals and reagents

UA (purity > 98%) and mebendazole (purity > 98%) were purchased from Aladdin (Shanghai, China) and Sigma-Aldrich (St. Louis, USA), respectively. These powders were prepared as a 10 mg/ml stock solution in DMSO for in vitro experiments. On the day of use, the stock solutions were diluted in DMSO to the desired concentration. For in vivo experiments, these two chemicals were suspended in 1% tragacanth solution and stored at 4 °C. Unless stated otherwise, all culture media and reagents were purchased from Gibco-BRL (Zurich, Switzerland).

### Parasites and animals

Liver hydatid cysts were obtained from sheep suffering from echinococcosis immediately after slaughter at abattoirs in Qinghai, China. The protoscoleces from the cysts were rinsed 5–8 times with PBS containing penicillin G (500 U/ml) and streptomycin (500 U/ml) for the collection of live parasites. Female Kunming (KM) mice (aged 4 weeks, 18–22 g) were purchased from the SLAC Laboratory Animal Center (Shanghai, China). Each mouse was inoculated intraperitoneally with 2000 protoscoleces. The genotype of protoscoleces from sheep and germinal cells from secondary infected mice was identified as *E. granulosus* G1 strain [20].

### In vitro culture of protoscoleces, germinal cells and metacestodes

Protoscoleces were maintained as previously described [20] with minor modifications. In brief, protoscoleces were cultured in RPMI 1640 medium supplemented with 10% FBS, 10% hydatid fluid, reducing agents (1 × 10^−5^ M L-dithiothreitol and 100 μM L-cysteine), 2 mM glutamine, 1 mM sodium pyruvate, 100 U/ml penicillin G, and 100 μg/ml streptomycin, at 37 °C under 5% CO_2_. The culture medium was changed every 4–5 days. Metacestodes were collected aseptically from the mice more than 10 months after secondary infection with *E. granulosus* protoscoleces. Only the cysts distributed in the abdominal cavity and not adhering to the tissues were selected for experiments. After being washed 4–5 times with PBS, metacestodes (diameter < 1 cm) were maintained using the same culture conditions as those used to maintain protoscoleces. The larger metacestodes were maintained in PBS for 24 h to exclude contamination with host cells. Then germinal cells were collected and cultured as previously reported [20]. The culture conditions were the same as those used for protoscoleces, although the medium was changed every 2–3 days.

### Confirmation of germinal cells by qPCR

Germinal cells (without host cell contamination) were characterized by qPCR as follows: RNA was extracted from *E. granulosus* germinal cells, *E. granulosus* protoscoleces (as positive control) and host tissues (normal mice liver) using RNeasy Mini Kit (Qiagen, Hilden, Germany) according to the manufacturer’s instructions. The first-strand cDNA was then synthesized from total RNA treated with DNase I (Thermo Fisher Scientific, California, USA) using the ReverTra Ace qPCR Kit (Toyobo, Osaka, Japan). PCR was performed using primers for the specific amplification of *E. granulosus* EF-1α [21] and mouse GADPH [22].

The qPCR reaction was performed using the C1000 Touch Thermal Cycler (Bio-rad, California, USA) in a final volume of 20 μl: 10 μl SYBR Green PCR Master Mix (Thermo Fisher Scientific), 0.8 μl of each primer (10 pmol/μl), 2 μl cDNA product, 6.4 μl ddH_2_O. Amplification was performed using the following conditions: 95 °C for 30 s and 40 cycles of denaturation at 95 °C for 5 s, annealing at 60 °C for 10 s and polymerization at 72 °C for 30 s. The PCR products were then heated to 95 °C for 10 s. A melting curve was created by cooling the products to 65 °C and then heating to 95 °C at a rate of 0.1 °C/s while measuring of the fluorescence simultaneously. PCR products were separated by 2% agarose gel electrophoresis and stained with Goodview (Beijing SBS Genetech, Beijing, China) before visualization under UV light.

### The effects of UA on protoscoleces, germinal cells and metacestodes in vitro

Protoscoleces (*n* = 50/well) or germinal cells (approximately 5 × 10^4^/well) were seeded in 96-well microtiter plates (Costar, Corning, USA) in 200 μl medium. Metacestodes (*n* = 6/well) were plated in 6-well microtiter plates (Costar) in 6 ml medium. UA was dissolved in DMSO and added to the medium at a final concentration of 1 to 40 μg/ml; 0.5% DMSO served as the control.

Protoscoleces and germinal cells were treated for 72 h. Metacestodes were treated by adding different concentrations of UA to the culture medium once each week for 2 weeks. Each experiment was repeated at least three times.

The viability of protoscoleces was determined using the methylene blue exclusion method as described previously [20]. In brief, 100 μl 0.1% methylene blue was added to each well. After 2 min, the protoscoleces were observed under an inverted microscope (dead protoscoleces were stained blue and the surviving ones remained colorless). The viability of germinal cells was tested using the Cell counting kit-8 (Dojindo, Kumamoto, Japan) according to the manufacturer’s instructions. The criteria for metacestode vitality was assessed on the basis of structural vesicle integrity.

### The efficacy of UA in experimentally infected mice

KM mice (*n* = 32) that had been infected with *E. granulosus* protoscoleces for 8 months were randomly divided into four groups (8 animals per group). These animals received UA (suspended in 1% tragacanth solution) via the oral route at 200 and 100 mg/kg/day (UA-200 and UA-100), and mebendazole (MBZ) at 25 mg/kg/day (MBZ-25) (also suspended in 1% tragacanth solution) for 28 consecutive days. The remaining group was treated orally with 1% tragacanth solution (1% TRA) as a control. During the treatment, mice were monitored daily for changes in behavior and body weight changes as well as survival. Two weeks after treatment, all mice were sacrificed by cervical dislocation, and the cysts in the peritoneal cavity were isolated and weighed. The efficacy of the treatment was assessed based on mean cyst weight [23] and ultrastructural changes in protoscoleces and germinal cells.

### Morphological and ultrastructural investigations of UA-treated protoscoleces, germinal cells and metacestodes

For investigations of the ultrastructure, germinal cells were grown on glass coverslips prior to drug treatment. Samples of protoscoleces, germinal cells and metacestodes cultured in vitro and treated with UA as well as cysts obtained from treated mice and their controls were processed for evaluation by scanning and transmission electron microscopy (SEM and TEM), respectively, as previously described [24].

### Cytotoxicity assay

Cytotoxicity was determined using three non-carcinogenic cell lines (L929 cells, HK-2 cells and Chang liver cells cultured in RPMI 1640, DMEM/F12 and DMEM, respectively) and three cancer cell lines (A2058 cells cultured in RPMI1640 and A172 cells and HCT-8 cells cultured in DMEM) (all cell lines were purchased from the Cell Bank of the Chinese Academy of Sciences); all media were supplemented with 10% FBS. The six cell types were seeded in 96-well microtiter plates (5 × 10^4^ cells/well). UA was added, and plates were incubated for 72 h at 37 °C under 5% CO_2_. Cell viability was determined with CCK-8 kits using the same method used to evaluate the viability of germinal cells. The effects of UA on non-carcinogenic cell lines were analyzed in the range 1.56–50.00 μg/ml, and on cancer cell lines in the range 0.39–12.5 μg/ml.

### Statistical analysis

The differences in mean cyst weights among groups were assessed by Kruskal-Wallis H-test and paired comparisons. IC_50_ (concentration that inhibited 50% germinal cell growth) and Tox_50_ (concentration that induced 50% cell toxicity) values were calculated using the probability unit method with SPSS version 17.0; *P* < 0.05 was considered to indicate statistical significance.

## Results

### The in vitro activity of UA against *E. granulosus* protoscoleces

**Fig. 1:**
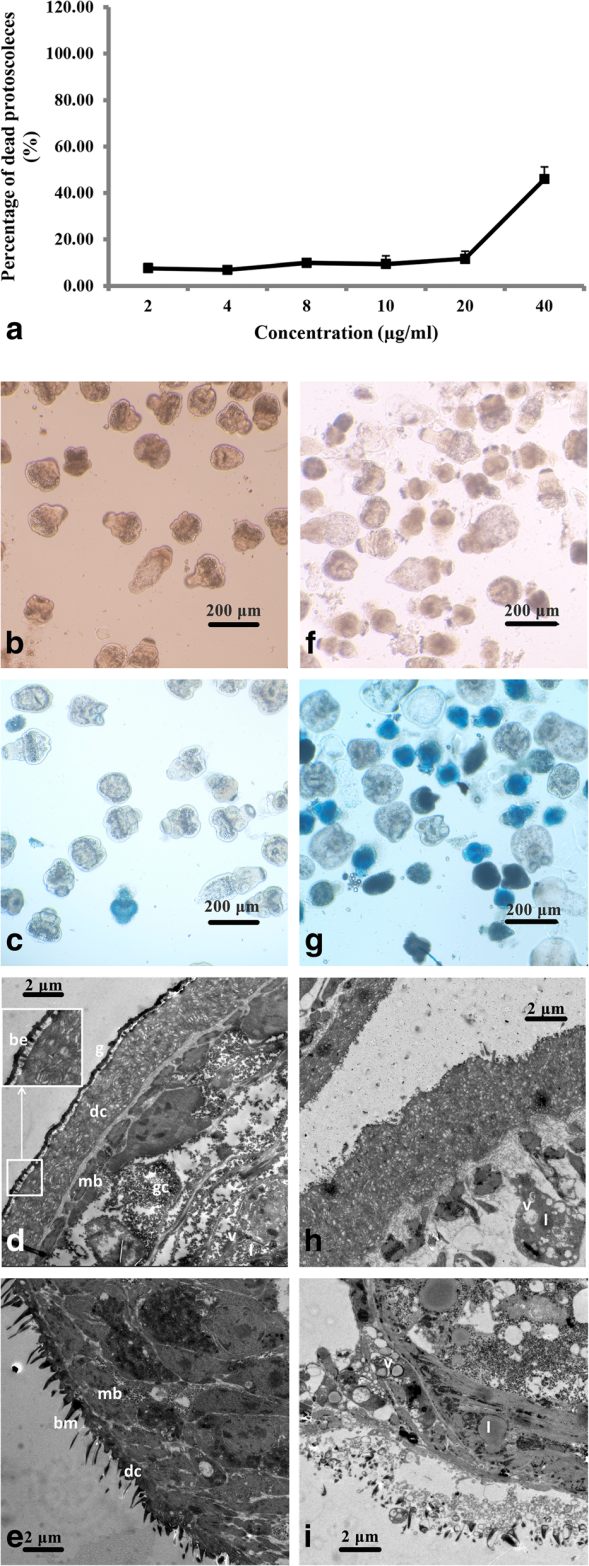
The effect of UA on *E. granulosus* protoscoleces cultured in vitro and related ultrastructural changes. **a** The viability of protoscoleces cultured in vitro with UA for 3 days was determined using the methylene blue exclusion method. The percentage of dead protoscoleces in the 0.5% DMSO control was 6.43 ± 1.00%; **b**-**e** Protoscoleces treated with 0.5% DMSO in vitro; **f-i** Protoscoleces cultured in vitro with UA at 40 μg/ml for 3 days. **b**, **f** Protoscoleces observed under light microscopy. **c**, **g** Stained protoscoleces observed under light microscopy. **d**, **h** The ultrastructure of the soma tegument of protoscoleces. **e**, **i** The ultrastructure of the rostellar tegument of protoscoleces. *Abbreviations*: g, glycocalyx; be, blunt elevation; dc, distal cytoplasm; mb, muscle bundles; gc, glycogen; v, vacuoles; l, lipid granules

After UA treatment at 40 μg/ml for 3 days, 45.95 ± 5.30% of protoscoleces were killed, while there was no significant effect on their viability at 20 μg/ml (Fig. 1a). Dead protoscoleces were distinguished from viable protoscoleces by the methylene blue exclusion method and visualized under light microscopy (Fig. 1b-g). The dead protoscoleces induced by UA treatment showed morphological changes, while micro-cysts were observed in the surviving protoscoleces. In contrast, no morphological changes were observed in the protoscoleces in the control group.

Ultrastructural damage was revealed by TEM in the protoscoleces treated with UA at 40 μg/ml for 3 days compared with the control protoscoleces cultured in 0.5% DMSO medium (which resulted in a death-rate of 6.43 ± 1.00%). No changes in ultrastructure were observed throughout the experimental period in the control group (Fig. 1d, e). In contrast, the soma tegument of protoscoleces appeared swollen with partial collapse and disruption of the inner structure following UA treatment. The distal cytoplasm at the rostellar and soma tegument was almost detached from its underlying basal matrix. Moreover, partial lysis of muscle cells and extracellular matrices accompanied by the formation of large vacuoles and lipid granules were also observed (Fig. 1h, i).

### The in vitro activity of UA against *E. granulosus* germinal cells

**Fig. 2:**
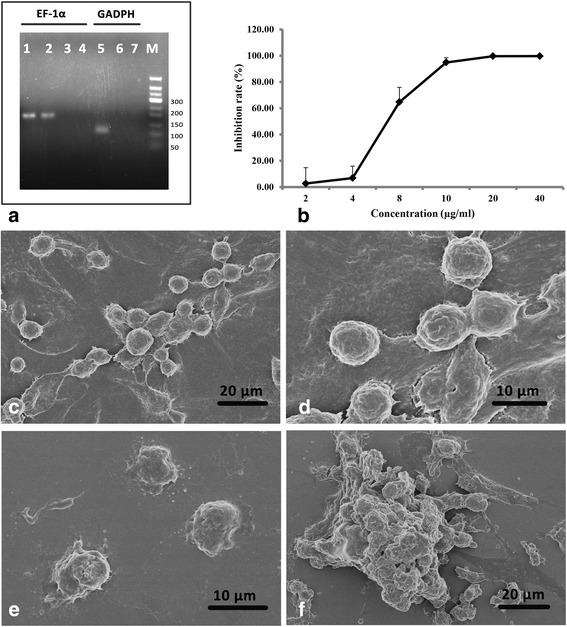
The effect of UA on *E. granulosus* germinal cells treated in vitro with UA. **a** *Echinococcus granulosus* EF-1α and mice GAPDH were used for the characterization of *E. granulosus* germinal cells (without host cell contamination). Lane 1: protoscoleces; Lanes 2 and 6: germinal cells; Lanes 3 and 5: mouse liver; Lanes 4 and 7: negative control. **b** The rate of germinal cell growth inhibition following treatment with UA in vitro. The data represent the mean ± standard deviation. **c**, **d** The ultrastructural changes of germinal cells cultured with 0.5% DMSO in vitro. **e**, **f** The ultrastructural changes of germinal cells treated with UA at 40 μg/ml for 3 days

In the qPCR analysis, there was no amplification of mouse GADPH in *E. granulosus* germinal cell culture (Ct = 24.03 ± 0.23), thus confirming the absence of host cells (Fig. 2a, Lane 6), while the *E. granulosus* EF-1α specific gene was amplified only from germinal cell culture (Ct = 28.00 ± 0.11) and protoscoleces (Ct = 24.13 ± 0.62).

Compared to the effects on *E. granulosus* protoscoleces, UA had a considerably stronger, and also dose-dependent, effect on germinal cells over 3 days. At 10, 20 and 40 μg/ml, UA resulted in a dramatic reduction of viable cells, with the inhibition rate higher than 90% (Fig. 2b). Moreover, germinal cell growth inhibition was reduced to 64.68 ± 11.22% by incubation with 8 μg/ml of UA and 3.76 ± 16.53% at 4 μg/ml (Fig. 2b) and the IC_50_ was calculated to be 5.65 ± 0.84 μg/ml. The effects of UA on *E. granulosus* germinal cells were also confirmed at the ultrastructural level by SEM (Fig. 2c-f). Germinal cells comprise a mixture of several cell types, including undifferentiated cells, muscle cells and glycogen storage cells. Most of germinal cells were round and adhered to the coverslips, exhibiting no ultrastructural alterations during the entire incubation period in comparison to the cells in the control group (Fig. 2c, d). In contrast, ultrastructural damage was detected in 40 μg/ml UA-treated germinal cells, which exhibited dramatic changes in morphology and the almost complete loss of protrusions and adherence filaments (Fig. 2e). Moreover, aggregation of dead cells was observed (Fig. 2f).

### The in vitro activity of UA against *E. granulosus* metacestodes

**Fig. 3:**
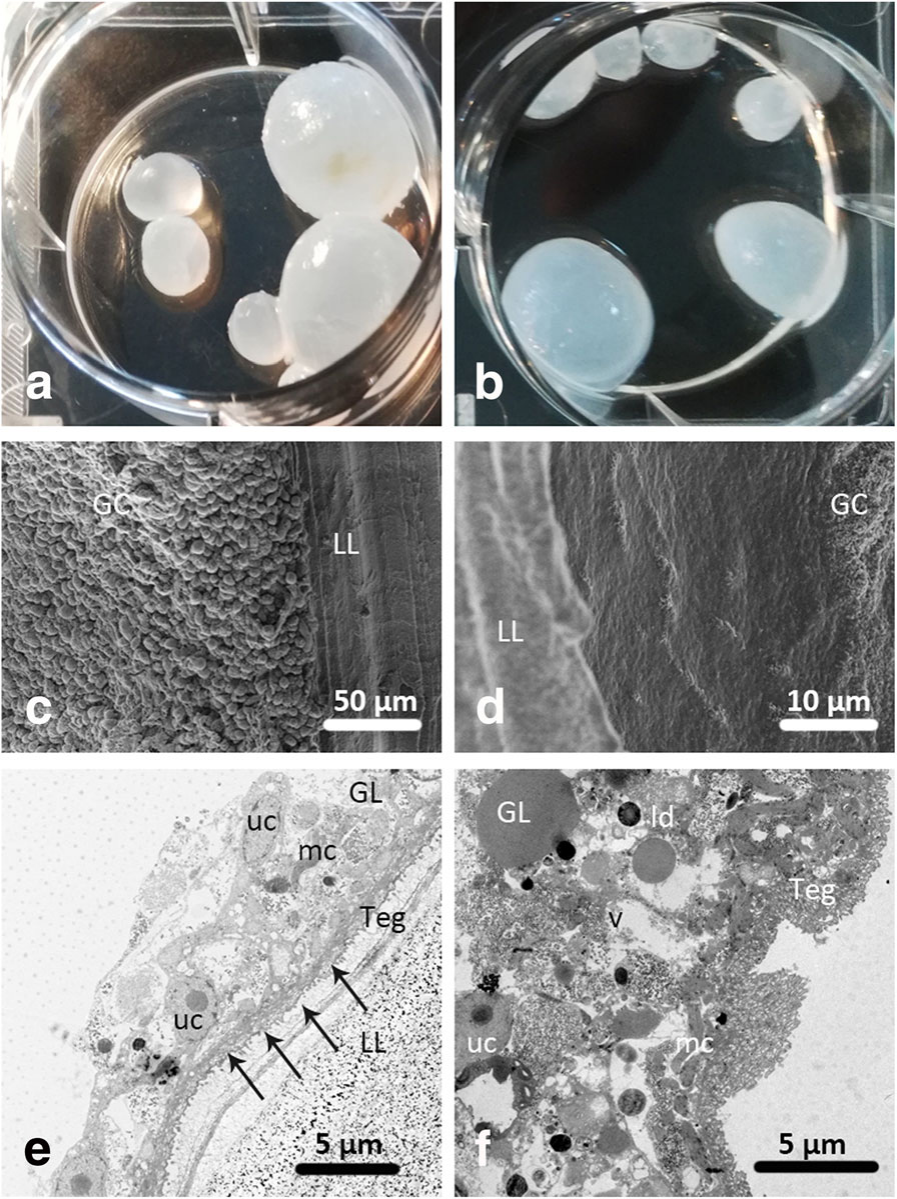
Cyst integrity, and ultrastructural changes of *E. granulosus* murine metacestodes incubated in vitro with UA at 40 μg/ml for 14 days. **a**, **c**, **e** 0.5% DMSO. **b**, **d**, **f** UA 40 μg/ml. Arrows indicate microtriches that protrude into the laminated layer. *Abbreviations*: LL, laminated layer; GL, germinal layer; Teg, tegument; uc, undifferentiated cells; mu, muscle cell; v, vacuoles; ld, lipid droplets

Untreated *E. granulosus* metacestodes obtained from in vitro cultures are basically fluid-filled cysts, with a distinct acellular outer laminated layer and an intact germinal layer. No alterations in *E. granulosus* metacestodes were observed after 14 days of incubation with 0.5% DMSO (Fig. 3a). However, the effect was detected in metacestodes treated with 10, 20 and 40 μg/ml of UA at 7 days after initiation of treatment. At the end of the experiment, more than 50% of metacestodes showed loss of integrity and typical changes, such as softening of the cysts and visible separation of the germinal and laminated layers (Fig. 3b). However, no effects on the morphology of metacestodes were observed following treatment with UA at 1 and 5 μg/ml for 14 days (Table 1).

**Table 1:** Time of appearance (days post-incubation) of tissue damage in *E. granulosus* murine metacestodes after their incubation with UA in vitro

UA (μg/ml)	No. of metacestodes that lost integrity (*n* = 6)^a^					
	Days post-incubation					
	1	4	7	9	12	14
40	0	0	1	2	5	5
20	0	0	1	1	4	4
10	0	0	1	2	3	3
5	0	0	0	0	0	0
1	0	0	0	0	0	0
0.5% DMSO as control	0	0	0	0	0	0

^**a**^Data shown are from one of three repeated experiments that produced similar results

SEM studies revealed that the germinal layer of UA-treated metacestodes lost its characteristic multicellular structure. Metacestodes treated with 0.5% DMSO exhibited an intact germinal layer with an abundance of cells (Fig. 3c), while the UA-treated germinal layer was largely detached in many areas, with only cellular debris remaining (Fig. 3d). TEM studies were conducted to provide more detailed information of the ultrastructure of metacestodes. Normally, the external surface of the parasite is surrounded by an acellular laminated layer and the parasite tissue attaches to the interior surface of the laminated layer via numerous microtriches. Furthermore, the tegument layer is located toward the interior followed by the germinal layer that contains numerous cell types. In this study, metacestodes treated with 0.5% DMSO for 14 days did not exhibit any ultrastructural changes, while UA treatment induced drastic destruction of parasites (Fig. 3e, f) and complete separation of the germinal layer from the laminated layer. In detail, the microtriches disappeared, the tegument layer was swollen, vacuolization increased and lipid droplets formed in the germinal layer (Fig. 3f).

### The in vivo efficacy of UA in experimentally infected mice

Intraperitoneally infected KM strain mice were exposed to different treatment regimens involving MBZ and UA for 4 weeks as indicated in Table 2. At the end of treatment, these animals were sacrificed by cervical dislocation and the total parasite weight was measured to calculate the reduction in cyst weight (Table 2). There was a significant difference among the four groups (*χ*^2^ = 17.769, *df* = 3, *P* < 0.001). Furthermore, daily oral administration of MBZ at 25 mg/kg significantly decreased the cyst weight (62.9%; *P* < 0.001). Oral administration of UA at 200 and 100 mg/kg also reduced the growth of the metacestodes in vivo (39.5%, *P* = 0.023 and 38.3%, *P* = 0.016, respectively). Furthermore, the cyst weight was significantly reduced in the MBZ-25 group compared with that in the UA-200 (*P* = 0.044) and UA-100 (*P* = 0.045) groups, although there was no significant difference between the UA-200 and UA-100 groups (*P* = 0.940).

**Table 2:** Effects of UA on mice infected with *E. granulosus* for 8 months (*n* = 8)

Group	Dose (× 28 days)	Mean cyst weight ± SD (g)	Mouse deaths during the experimental period	Cyst weight reduction (%)
Control (1% tragacanth)	0.1 ml/10 g/day	19.73 ± 17.22	2	–
Mebendazole	25 mg/kg/day	7.33 ± 3.67*	1	62.9
Ursolic acid	200 mg/kg/day	11.94 ± 5.69*	1	39.5
	100 mg/kg/day	12.18 ± 8.64*	0	38.3

*Indicates that there were significant reductions in parasite weight achieved by treatment with mebendazole at 25 mg/kg and UA at 200 mg/kg and 100 mg/kg compared that achieved by treatment with 1% tragacanth

*Abbreviation*: SD, standard deviation

**Fig. 4:**
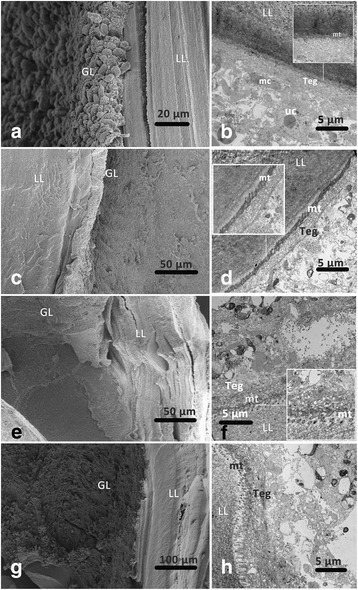
Ultrastructural changes of hydatid cysts with different treatments in vivo. **a**, **b** 1% TRA as control. **c**, **d** MBZ 25 mg/kg. **e**, **f** UA 200 mg/kg. **g**, **h** UA 100 mg/kg. *Abbreviations*: LL, laminated layer; GL, germinal layer;Teg, tegument; uc, undifferentiated cells; mu, muscle cell; mt, microtriches

TEM and SEM studies of metacestodes isolate from mice following treatment with UA revealed extensive parasite tissue damage (Fig. 4). Hydatid cysts from infected mice treated with 1% TRA presented intact structural features, such as a well-defined laminated layer, an intact and densely-packed germinal layer, protrusion of the microtriches into the laminated layer, and abundant undifferentiated cells and muscle cells (Fig. 4a, b). SEM studies revealed distinct changes in metacestodes from MBZ- and UA-treated mice, including detachment of the germinal layer from the laminated layer (Fig. 4e) and loss of the normal structure of germinal layer (Fig. 4c, g). Following treatment, TEM studies showed marked destruction of the germinal layer characterized by the absence of cellular structures, together with the formation of vacuoles in the tegument and swelling or destruction of microtriches that were partially separated from the laminated layer (Fig. 4c–h).

### Cytotoxicity of UA in non-carcinogenic cells and cancer cell lines

The cytotoxicity of UA was assessed in mammalian non-carcinogenic cell lines (L929, HK-2 and Chang liver) and cancer cell lines (A172, A2058 and HCT-8) using the CCK-8 method. The Tox_50_ values for the three non-carcinogenic cell lines were 13.69 ± 1.39, 11.41 ± 2.24 and 13.97 ± 1.67 μg/ml, respectively. In contrast, the cancer cells were more sensitive to UA, with Tox_50_ values of 2.54 ± 0.20, 2.68 ± 0.04 and 2.80 ± 0.02 μg/ml, respectively.

## Discussion

Since drug research and development is time-consuming and expensive, a drug repurposing strategy offers an alternative route to significantly shorten the traditional drug development process [25]. In the present study, we investigated the in vitro anthelmintic effects of UA on protoscoleces, germinal cells and metacestodes of *E. granulosus* and the in vivo efficacy in experimentally infected mice. Although treatment with 40 μg/ml UA for 3 days only resulted in the death of 45.95 ± 5.30% protoscoleces, the survival of germinal cells was found to be inhibited in a dose-dependent manner, with > 90% inhibition at 10, 20 and 40 μg/ml. These results indicated that germinal cells were more sensitive to UA than protoscoleces. This information has important implication for the potential treatment of echinococcosis, because germinal cells are the only proliferative part of the parasite and are usually considered as the main target for chemotherapy [26]. In addition, UA was also shown to be effective on cultured metacestodes in vitro. In the infected organs (especially the liver and lungs) of hosts, these cysts have a basic bladder-like morphology and are protected by the extracellular carbohydrate-rich laminated layer (LL), which is synthesized by the underlying cellular germinal layer (GL) [27]. Our results confirmed the ability of UA at effective concentrations to cross the LL to induce the death of the parasites. In our in vitro study, MBZ was used as the positive control and 0.5% DMSO was used as the negative control since this is the final concentration of DMSO required for complete dissolution of MBZ. The final results indicated that the negative control did not influence the activity of parasites during the observation period.

To test the in vivo efficacy, UA was administered at 200 and 100 mg/kg for the treatment of echinococcosis according to the regimen adopted for the treatment of cancers with UA [11, 28]. After treatment for 28 days, reduction in growth and cyst weight as well as loss of metacestode integrity was found, and this effect was significant in the groups treated with 200 mg/kg and 100 mg/kg of UA. Furthermore, the toxicity of UA was also discussed in our study. To properly compare the parasite and mammalian cells, the same viability assay was applied by CCK-8 kit. For three mammalian normal cell lines, the Tox_50_ was calculated to be 11–14 μg/ml, which was 2- to 3-fold in excess of the measured IC_50_ values observed for germinal cells. These results proved that UA was more effective against parasites than normal host cells, but also indicated a potential safety concern. Hence, during the 28 days of treatment, we paid close attention to the conditions of the animals. However, no mice died during the experiments, while two mice and one mouse died in the control and MBZ-25 groups, respectively, which preliminarily indicated that the use of UA for the treatment of murine-echinococcosis was safe in our conditions. In addition, the regimen used in this study has been proved to be safe in the treatment of parasites and cancer in animals and humans [8, 18]. Still, considering long-term chemotherapy for echinococcosis, scientific and objective safety and toxicity of UA must be evaluated in future.

The efficacy of UA against echinococcosis in the murine model might be attributed to its direct effect on the parasite, as well as its impact on host according to the mechanisms of UA against cancers. It was reported that UA can inhibit cell growth, migration and invasion in many cancer cell models and exert antiproliferative effects [8] via several mechanisms that are also important for the development and survival of *Echinococcus* spp. For example, UA was reported to induce apoptosis through suppression of the components of the ERK/MAPK signaling pathway [29, 30], which plays important roles in the growth and development of *Echinococcus* spp*.* [31, 32]. Human tumor suppressor p53 was effected by UA, and its homologue-Emp53 in *E. multilocularis* was reported to play a functional role in the *E. multilocularis* metacestode [33]. In addition to the induction of apoptosis, UA has also been reported to prevent the metastasis of cancer through down-expression of matrix metalloproteinases (MMP-2 and MMP-9) [12, 34] relating to the microenvironment of cancer cells; these enzymes were detected in *E. granulosus* hydatid cyst fluid, cyst membranes and protoscolices, and are involved in pathology-related tissue remodeling [35, 36].

Poor solubility of UA in aqueous solutions greatly limits its application [13]. In the treatment of several cancers, no differences of efficacy were shown between 50 mg/kg/d and 200 mg/kg/d of UA [37, 38]. In our study, similar therapeutic outcomes of the two UA groups were also found. Hence, because of its poor water solubility and low bioavailability, UA has been administered using liposomes [39, 40]. This characteristic of UA indicates that the appropriate formulation of UA could potentially enhance the treatment efficacy against echinococcosis.

## Conclusions

The findings of the present study demonstrate the efficacy and parasiticidal activity of UA against *E. granulosus* protoscoleces, germinal cells and metacestodes in vitro and in vivo. Thus, UA is implicated as a potential anti-echinococcosis agent. However, its mechanism and the application prospects with proper formulation remain to be investigated.

## Abbreviations

CCK-8: cell counting kit-8
CE: Cystic echinococcosis
GL: Germinal layer
KM mouse: Kunming mouse
LL: Laminated layer
MBZ: Mebendazole
SEM: Scanning electron microscopy
TEM: Transmission electron microscopy
UA: Ursolic acid
WHO: World Health Organization

## References

[1] Eckert J, Thompson RC (2017). Historical aspects of echinococcosis. Adv Parasitol.

[2] Thompson RC (2008). The taxonomy, phylogeny and transmission of *Echinococcus*. Exp Parasitol.

[3] Eckert J, Pawlowski Z, Dar FK, Vuitton DA, Kem P, Savioli L (1995). Medical aspects of echinococcosis. Parasitol Today.

[4] Nicolao MC, Elissondo MC, Denegri GM, Goya AB, Cumino AC (2014). In vitro and in vivo effects of tamoxifen against larval stage *Echinococcus granulosus*. Antimicrob Agents Chemother.

[5] Stadelmann B, Aeschbacher D, Huber C, Spiliotis M, Muller J, Hemphill A (2014). Profound activity of the anti-cancer drug bortezomib against *Echinococcus multilocularis* metacestodes identifies the proteasome as a novel drug target for cestodes. PLoS Negl Trop Dis.

[6] Pensel PE, Albani C, Gamboa GU, Benoit JP, Elissondo MC (2014). In vitro effect of 5-fluorouracil and paclitaxel on *Echinococcus granulosus* larvae and cells. Acta Trop.

[7] Hemer S, Brehm K (2012). In vitro efficacy of the anticancer drug imatinib on *Echinococcus multilocularis* larvae. Int J Antimicrob Agents.

[8] Wozniak L, Skapska S, Marszalek K (2015). Ursolic acid - a pentacyclic triterpenoid with a wide spectrum of pharmacological activities. Molecules.

[9] Shanmugam MK, Dai X, Kumar AP, Tan BK, Sethi G, Bishayee A (2013). Ursolic acid in cancer prevention and treatment: molecular targets, pharmacokinetics and clinical studies. Biochem Pharmacol.

[10] Shao JW, Dai YC, Xue JP, Wang JC, Lin FP, Guo YH (2011). In vitro and in vivo anticancer activity evaluation of ursolic acid derivatives. Eur J Med Chem.

[11] De Angel RE, Smith SM, Glickman RD, Perkins SN, Hursting SD (2010). Antitumor effects of ursolic acid in a mouse model of postmenopausal breast cancer. Nutr Cancer.

[12] Huang CY, Lin CY, Tsai CW, Yin MC (2011). Inhibition of cell proliferation, invasion and migration by ursolic acid in human lung cancer cell lines. Toxicol in Vitro.

[13] Qian Z, Wang X, Song Z, Zhang H, Zhou S, Zhao J (2015). A phase I trial to evaluate the multiple-dose safety and antitumor activity of ursolic acid liposomes in subjects with advanced solid tumors. Biomed Res Int.

[14] do Nascimento PG, Lemos TL, Bizerra AM, Arriaga AM, Ferreira DA, Santiago GM (2014). Antibacterial and antioxidant activities of ursolic acid and derivatives. Molecules.

[15] Bag P, Chattopadhyay D, Mukherjee H, Ojha D, Mandal N, Sarkar MC (2012). Anti-herpes virus activities of bioactive fraction and isolated pure constituent of *Mallotus peltatus*: an ethnomedicine from Andaman Islands. Virol J.

[16] van Baren C, Anao I, Leo Di Lira P, Debenedetti S, Houghton P, Croft S (2006). Triterpenic acids and flavonoids from *Satureja parvifolia*. Evaluation of their antiprotozoal activity. Z Naturforsch C.

[17] Cunha NL, Uchoa CJ, Cintra LS, de Souza HC, Peixoto JA, Silva CP, et al. In vitro schistosomicidal activity of some Brazilian Cerrado species and their isolated compounds. Evid Based Complement Alternat Med. 2012;2012:173614. 10.1155/2012/173614 PMC3424599 22924053

[18] Kalani K, Kushwaha V, Sharma P, Verma R, Srivastava M, Khan F (2014). In vitro, in silico and in vivo studies of ursolic acid as an anti-filarial agent. PLoS One.

[19] Vijaya YAK. In vitro anthelmintic assessment of selected phytochemicals against *Hymenolepis diminuta*, a zoonotic tapeworm. J Parasit Dis. 2016;40(3):1082–6. 10.1007/s12639-014-0560-1 PMC4996173 27605841

[20] Liu C, Zhang H, Yin J, Hu W (2015). In vivo and in vitro efficacies of mebendazole, mefloquine and nitazoxanide against cyst echinococcosis. Parasitol Res.

[21] Espinola SM, Ferreira HB, Zaha A. Validation of suitable reference genes for expression normalization in *Echinococcus *spp. larval stages. PLoS One. 2014;9(7):e102228. 10.1371/journal.pone.0102228 PMC4094502 25014071

[22] Fragkouli A, Papatheodoropoulos C, Georgopoulos S, Stamatakis A, Stylianopoulou F, Tsilibary EC (2012). Enhanced neuronal plasticity and elevated endogenous sAPPalpha levels in mice over-expressing MMP9. J Neurochem.

[23] Liu CS, Zhang HB, Jiang B, Yao JM, Tao Y, Xue J, et al. Enhanced bioavailability and cysticidal effect of three mebendazole-oil preparations in mice infected with secondary cysts of *Echinococcus granulosus*. Parasitol Res 2012;111(3):1205–11. 10.1007/s00436-012-2954-2 22661241

[24] Liu C, Yin J, Xue J, Tao Y, Hu W, Zhang H. In vitro effects of amino alcohols on *Echinococcus granulosus*. Acta Trop. 2017. 10.1016/j.actatropica.2017.08.031. 10.1016/j.actatropica.2017.08.031 28859963

[25] Klug DM, Gelb MH, Pollastri MP (2016). Repurposing strategies for tropical disease drug discovery. Bioorg Med Chem Lett.

[26] Eckert J, Gemmell MA, Meslin FX (2001). WHO/OIE manual on echinococcosis in humans and animals: a public health problem of global concern.

[27] Diaz A, Casaravilla C, Allen JE, Sim RB, Ferreira AM (2011). Understanding the laminated layer of larval *Echinococcus* II: immunology. Trends Parasitol.

[28] Shih WL, Yu FL, Chang CD, Liao MH, Wu HY, Lin PY (2013). Suppression of AMF/PGI-mediated tumorigenic activities by ursolic acid in cultured hepatoma cells and in a mouse model. Mol Carcinog.

[29] Li Y, Lu X, Qi H, Li X, Xiao X, Gao J (2014). Ursolic acid induces apoptosis through mitochondrial intrinsic pathway and suppression of ERK1/2 MAPK in HeLa cells. J Pharmacol Sci.

[30] Shan JZ, Xuan YY, Zheng S, Dong Q, Zhang SZ (2009). Ursolic acid inhibits proliferation and induces apoptosis of HT-29 colon cancer cells by inhibiting the EGFR/MAPK pathway. J Zhejiang Univ Sci B.

[31] Gelmedin V, Spiliotis M, Brehm K (2010). Molecular characterisation of MEK1/2- and MKK3/6-like mitogen-activated protein kinase kinases (MAPKK) from the fox tapeworm *Echinococcus multilocularis*. Int J Parasitol.

[32] Spiliotis M, Konrad C, Gelmedin V, Tappe D, Bruckner S, Mosch HU (2006). Characterisation of EmMPK1, an ERK-like MAP kinase from *Echinococcus multilocularis* which is activated in response to human epidermal growth factor. Int J Parasitol.

[33] Cheng Z, Zhu S, Wang L, Liu F, Tian H, Pengsakul T (2015). Identification and characterisation of Emp53, the homologue of human tumor suppressor p53, from *Echinococcus multilocularis*: its role in apoptosis and the oxidative stress response. Int J Parasitol.

[34] Kim ES, Moon A (2015). Ursolic acid inhibits the invasive phenotype of SNU-484 human gastric cancer cells. Oncol Lett.

[35] Marco M, Baz A, Fernandez C, Gonzalez G, Hellman U, Salinas G (2006). A relevant enzyme in granulomatous reaction, active matrix metalloproteinase-9, found in bovine *Echinococcus granulosus* hydatid cyst wall and fluid. Parasitol Res.

[36] Marco M, Nieto A (1991). Metalloproteinases in the larvae of *Echinococcus granulosus*. Int J Parasitol.

[37] Hsu HY, Yang JJ, Lin CC (1997). Effects of oleanolic acid and ursolic acid on inhibiting tumor growth and enhancing the recovery of hematopoietic system postirradiation in mice. Cancer Lett.

[38] Shanmugam MK, Rajendran P, Li F, Nema T, Vali S, Abbasi T (2011). Ursolic acid inhibits multiple cell survival pathways leading to suppression of growth of prostate cancer xenograft in nude mice. J Mol Med (Berl).

[39] Yang G, Yang T, Zhang W, Lu M, Ma X, Xiang G (2014). In vitro and in vivo antitumor effects of folate-targeted ursolic acid stealth liposome. J Agric Food Chem.

[40] Both DM, Goodtzova K, Yarosh DB, Brown DA (2002). Liposome-encapsulated ursolic acid increases ceramides and collagen in human skin cells. Arch Dermatol Res.

